# Comparative genetic diversity of the endangered, narrowly endemic Chinese orchid *Bulbophyllum tianguii* and its widespread congener *Bulbophyllum andersonii*

**DOI:** 10.3389/fpls.2026.1857744

**Published:** 2026-07-13

**Authors:** Lihui Peng, Lingzhi Wei, Zhe Yang, Yishan Yang, Chenghao Zhu, Feng Chen, Yajin Luo, Zhenhai Deng, Xiao Wei, Shengfeng Chai

**Affiliations:** 1 Guangxi Institute of Botany, Guangxi Zhuang Autonomous Region and Chinese Academy of Sciences, Guilin, Guangxi, China; 2 Guangxi Key Laboratory of Plant Functional Substances and Sustainable Utilization, Guangxi Institute of Botany, Guilin, Guangxi, China; 3 Guilin University of Technology, Guilin, Guangxi, China; 4 Beijing Forestry University, Beijing, China; 5 Yachang Orchid National Nature Reserve Management Center, Baise, Guangxi, China

**Keywords:** *Bulbophyllum andersonii*, endangered plants, genetic diversity, genotyping-by-sequencing (GBS), Bulbophyllum tianguii, population structure, gene flow

## Abstract

**Introduction:**

Genetic research on endangered plant species is crucial for formulating effective biodiversity conservation and management strategies. *Bulbophyllum tianguii*, a rare and endangered orchid species endemic to the karst regions of Southwest China, possesses exceptionally high ornamental value. However, its population genetic structure and evolutionary potential remain largely unexplored. Therefore, this study aimed to comparatively evaluate the genetic diversity and population structure of the narrowly distributed *B. tianguii* and its widespread congener, *Bulbophyllum andersonii*.

**Methods:**

We utilized Genotyping-by-Sequencing (GBS) technology to discover 23,880 highquality single nucleotide polymorphisms (SNPs) across 50 *B. tianguii* (from seven populations) and 31 *B. andersonii* (from four populations) individuals.

**Results:**

The results revealed that the key genetic diversity parameters of *B. tianguii* populations (Ho = 0.101, He = 0.070, I = 0.088, π = 0.077) were significantly (P < 0.01) lower than those of B. andersonii (Ho = 0.105, He = 0.101, I = 0.183, π = 0.111). Population structure, phylogenetic tree, and Principal Component Analysis (PCA) consistently divided *B. tianguii* into six subpopulations, whereas *B. andersonii* comprised two subpopulations and one admixed group. Kinship and gene flow analyses indicated that *B. tianguii* exhibited weaker intra- and inter-population relatedness, accompanied by higher genetic differentiation (FST = 0.450) and restricted gene flow (proxy Nm = 0.411) compared to *B. andersonii* (FST = 0.268, proxy Nm = 0.830). Mantel tests confirmed that genetic differentiation was not significantly correlated with geographic distance for either species (P < 0.05).

**Discussion:**

Despite the limitations of lacking a reference genome and uneven sampling sizes, these findings suggest that *B. tianguii* is characterized by dangerously low genetic diversity. To preserve its gene pool, future conservation should prioritize in situ protection and careful reintroduction, while monitoring the potential risks of outbreeding depression.

## 1 Introduction

Genetic diversity is a fundamental component of biodiversity, referring to the total genetic variation among individuals within a species or population (Frankham et al., 2012**;** Allendorf et al., 2013). Population genetic structure describes the spatiotemporal distribution of this diversity, encompassing intra-population variation and inter-population differentiation. These factors are closely linked to a species’ environmental adaptability and evolutionary potential (Frankham et al., 2012**;** Li et al., 2020). Accurate assessment of genetic diversity and population structure is essential for formulating effective conservation strategies for endangered species (Teixeira and Nazareno, 2021). Narrowly distributed species typically exhibit lower genetic diversity compared to widespread species (Gitzendanner and Soltis, 2000**;** Gong et al., 2010). However, genetic diversity is influenced not only by distribution range but also by various life-history traits, such as breeding systems, seed and pollen dispersal mechanisms, and habitat characteristics (Nybom, 2004). This makes direct comparisons of genetic diversity between widespread and narrowly distributed species under a single standard challenging (Soto et al., 2016**;** Lemon and Wolf, 2018**;** Wu et al., 2020). Nevertheless, comparative studies using a closely related, widespread congener with similar life-history traits and reproductive mechanisms as a control have proven to be an effective approach. Such comparisons are crucial for elucidating the causes of endangerment and establishing a foundation for effective conservation and management strategies (Zhu et al., 2023).

*Bulbophyllum tianguii* (Orchidaceae), a narrow endemic species, is a rare and endangered orchid exclusive to China. It possesses exceptional ornamental and scientific value due to its striking, elegantly curled golden-yellow petals (Lang and Luo, 2007). This species predominantly inhabits the surfaces of karst limestone or humus-rich tree trunks in western Guangxi and southwestern Guizhou. Discovered as a new species in 2005 within the Guangxi Yachang Orchid National Nature Reserve, it is restricted to only four known locations with fewer than 2,000 individuals in the reserve. Although subsequently found in other locations (e.g., Wangmo Cycad Nature Reserve, Guizhou; Liuguang River in Xiuwen; and Nanmudu in Kaiyang), its total population size remains unquantified but extremely small and fragmented (Jiang et al., 2020). This species was assessed as Data Deficient (DD) in the China Biodiversity Red List: Higher Plants (2020). However, based on our recent field surveys, it possesses an extremely narrow distribution range and a weak natural reproductive capacity. Exacerbated by severe anthropogenic disturbances to its wild habitats in recent years, the species is currently endangered and has been included in the List of Key Protected Wild Plants in Guangxi Zhuang Autonomous Region. Therefore, there is an urgent need to conduct in-depth conservation genetic research on *B. tianguii*. Conversely, *Bulbophyllum andersonii* is a widespread congener within the same genus, characterized by a large population size, robust self-propagation ability, and strong environmental adaptability. It is widely distributed across Guangxi, Sichuan, Guizhou, and Yunnan in China, as well as in Sikkim, India, Myanmar, and Vietnam, typically thriving on tree trunks or rocks in mountainous forests at altitudes of 400–2,000 m (Zhao et al., 2013). Field investigations revealed sympatric distributions of both species in certain habitats, yet the individual count of *B. andersonii* consistently and significantly outnumbers that of *B. tianguii*.

To date, research on *B. tianguii* has been limited to preliminary studies focusing on pollination biology (Jiang et al., 2020**;** Jiang et al., 2025), associated community characteristics (Chai et al., 2022), photosynthetic physiology (Xiao, 2023), and mycorrhizal fungi (Liang et al., 2022). However, the genetic diversity and population structure of *B. tianguii* remain largely unexplored. Molecular markers, unaffected by environmental factors, are reliable tools for evaluating genetic diversity (Nadeem et al., 2017). Single Nucleotide Polymorphism (SNP), the most abundant type of sequence variation, are highly suitable for such analyses (Kumar et al., 2012). Advances in next-generation sequencing, particularly Genotyping-by-Sequencing (GBS), have facilitated cost-effective and high-throughput SNP discovery without requiring deep whole-genome coverage (He et al., 2014). GBS has been successfully applied in genetic mapping, phylogenetic analysis, and genetic diversity assessments across various plants (He et al., 2014**;** Elshire et al., 2011), including *Geodorum eulophioides* (Zhu et al., 2023), rice (Vasumathy et al., 2020), *Avena sterilis* (Al-hajaj et al., 2018), *Camellia sinensis* (Niu et al., 2019), and *Triticum turgidum* (Marcotuli et al., 2017). Given the large and complex genomes characteristic of many orchids (Yang et al., 2021**;** Piet et al., 2022), obtaining whole-genome SNPs via whole-genome resequencing remains economically and technically challenging; thus, GBS emerges as an optimal, cost-efficient strategy. Therefore, this study utilized GBS technology to discover genome-wide SNPs in both *B. tianguii* and *B. andersonii*. Based on these markers, we systematically analyzed and compared their genetic diversity, population structure, genetic differentiation, and gene flow.

Furthermore, epiphytic orchids often exhibit unique population genetic structures due to their fragmented habitats and specialized reliance on specific mycorrhizal fungi for seed germination (Phillips et al., 2012**;** McCormick and Jacquemyn, 2014). Both species share sympatric distributions in certain habitats and exhibit similar epiphytic/lithophytic growth habits, making *B. andersonii* an ideal comparative model despite some differences in specific life-history traits. We hypothesized that due to its intense karst habitat specificity and extremely small population size, *B. tianguii* would exhibit significantly lower genome-level genetic diversity compared to its widespread congener, *B. andersonii*. Investigating the population genetic dynamics of *B. tianguii* will help reveal its adaptive potential to environmental changes, identify its primary threatening factors, and ultimately provide a critical theoretical foundation for the effective conservation and scientific management of this rare, endemic orchid.

## 2 Materials and methods

### 2.1 Plant materials

**Figure 1:**
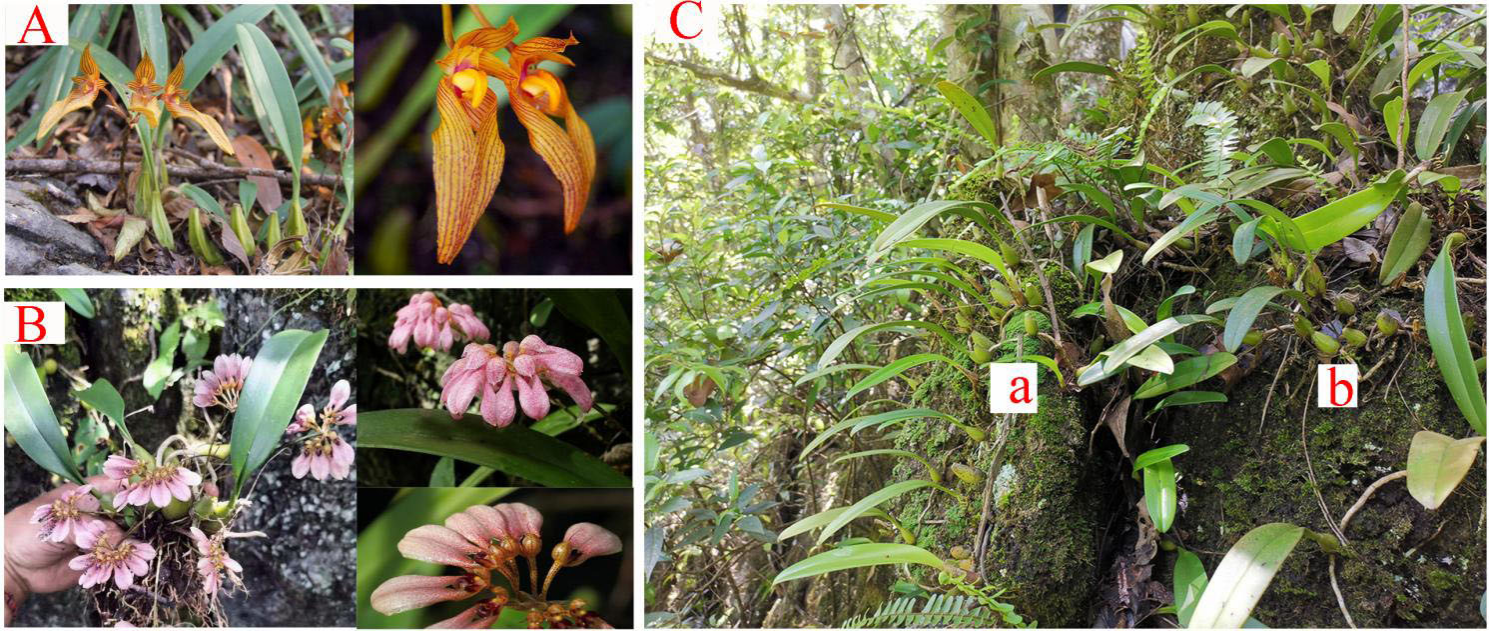
Schematic diagram of *B. tianguii* and *B. andersonii* [**(A)** *B. tianguii*; **(B)** *B. andersonii*; **(C)** Sympatric distribution of *B. andersonii* **(a)** and *B. tianguii* **(b)**].

**Figure 2:**
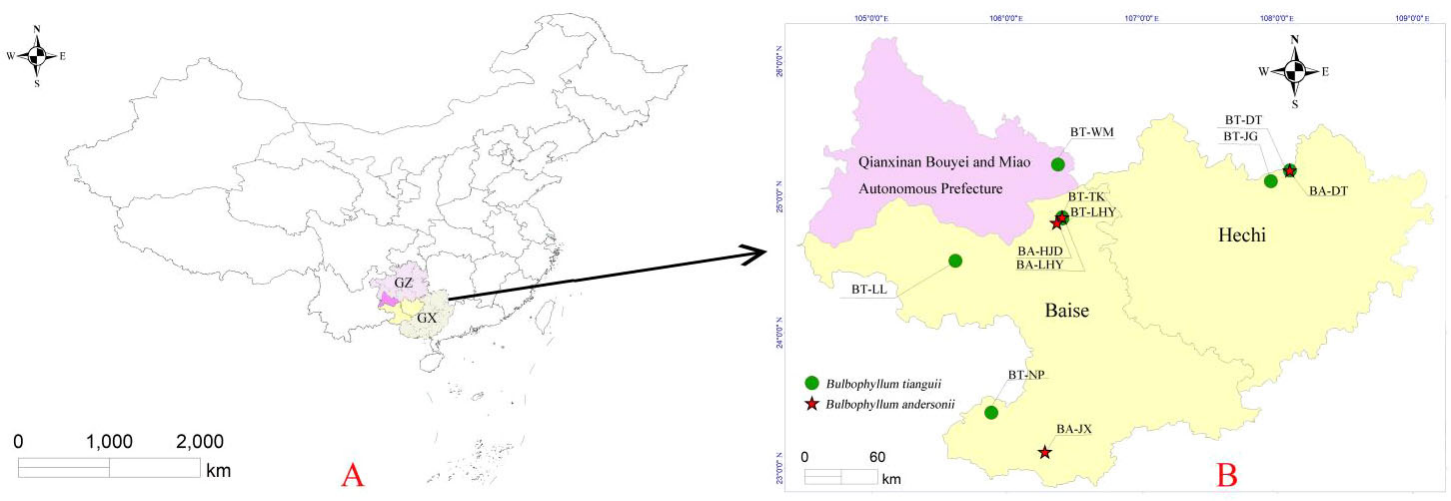
Geographical locations of the sampling sites for *B. tianguii* and *B. andersonii* in Guangxi and Guizhou [**(A)** Location of Guangxi and Guizhou in China; **(B)** Specific sampling distribution sites of the two species].

In 2024, a total of 81 plant samples from two species were collected from wild populations. These included 50 samples from seven populations of *B. tianguii* and 31 samples from four populations of *B. andersonii* (**Figures 1**, **2**; **Table 1**). The sampling sites were located across Baise City (Leye, Longlin, Napo, and Jingxi Counties) and Hechi City (Huanjiang County) in the Guangxi Zhuang Autonomous Region, as well as the Qianxinan Buyei and Miao Autonomous Prefecture (Wangmo County) in Guizhou Province. Two fresh, healthy, and young leaves were collected from each plant and immediately stored in sealed bags containing a sufficient amount of color-indicating silica gel for rapid desiccation. Each sample was carefully labeled. A minimum distance of 1–5 m was maintained between sampled individuals to avoid collecting clones. The GPS coordinates of each sampling site were recorded to calculate the geographic distances between populations based on latitude and longitude (**Table 2**). During the field surveys, sympatric distribution of *B. tianguii* and *B. andersonii* was observed at two specific sites: the Orchid Garden of the Yachang Nature Reserve and the Dongtian site at the Xiazhai Station of the Mulun National Nature Reserve in Guangxi (**Figure 1C**). All plant specimens were formally authenticated by Researcher Shengfeng Chai from the Guangxi Institute of Botany, Chinese Academy of Sciences. Finally, the desiccated samples were sent to Shanghai Lingen Biotechnology Co., Ltd. for reduced-representation genome sequencing.

**Table 1:** Sample collection information for *B. tianguii* and *B. andersonii*.

Species	Population	Collection location	Altitude (m)	Longitude (°E)	Latitude (°N)	Number of samples (individuals)	Total population (individuals)	Distribution area (m²)
*B. tianguii*	BT-LHY	Orchid Garden, Yachang Nature Reserve, Leye County, Baise City, Guangxi	950	106.31	24.85	9	125	12
	BT-TK	Laowuji Tiankeng (Sinkhole), Yachang Nature Reserve, Leye County, Baise City, Guangxi	1281	106.24	24.50	8	427	15
	BT-DT	Dongtian, Xiazhai Station, Mulun Nature Reserve, Huanjiang County, Hechi City, Guangxi	555	107.96	25.16	6	25	1
	BT-JG	Jian’gei, Mulun Station, Mulun Nature Reserve, Huanjiang County, Hechi City, Guangxi	739	107.93	25.18	7	615	56
	BT-LL	Jieting Township, Longlin County, Baise City, Guangxi	1147	105.37	24.65	6	320	42
	BT-NP	Chengxiang Town, Napo County, Baise City, Guangxi	1294	105.83	23.40	7	125	22
	BT-WM	Mashan Town, Wangmo County, Qianxinan Buyei and Miao Autonomous Prefecture, Guizhou	1245	106.26	25.26	7	225	28
*B. andersonii*	BA-LHY	Orchid Garden, Yachang Nature Reserve, Leye County, Baise City, Guangxi	950	106.31	24.85	8	850	50
	BA-HJD	Huangjingdong, Yachang Nature Reserve, Leye County, Baise City, Guangxi	1041	106.34	24.48	8	1500	120
	BA-DT	Dongtian, Xiazhai Station, Mulun Nature Reserve, Huanjiang County, Hechi City, Guangxi	555	107.96	25.16	8	1050	75
	BA-JX	Jiang (or Lutong Town), Jingxi County, Baise City, Guangxi	848	106.26	23.15	7	1200	90

**Table 2:** Geographic distances among the populations of *B. tianguii* and *B. andersonii* (Unit: km).

Distance	BT-LHY	BT-TK	BT-DT	BT-JG	BT-LL	BT-NP	BT-WM	BA-HJD	BA-LHY	BA-DT	BA-JX
BT-LHY	0.00										
BT-TK	1.09	0.00									
BT-DT	174.02	174.13	0.00								
BT-JG	158.42	158.49	16.67	0.00							
BT-LL	87.62	87.31	260.59	244.61	0.00						
BT-NP	168.94	167.96	299.36	282.92	127.39	0.00					
BT-WM	43.27	44.37	172.76	159.20	110.03	209.52	0.00				
BA-HJD	0.56	1.09	174.02	158.42	87.62	168.94	43.27	0.00			
BA-LHY	6.11	5.46	179.05	163.33	82.09	163.34	47.72	6.11	0.00		
BA-DT	174.02	174.13	0.00	16.67	260.59	299.36	172.76	174.02	179.05	0.00	
BA-JX	192.89	191.82	295.10	279.40	170.54	51.77	235.81	192.89	188.10	295.10	0.00

### 2.2 Methods

#### 2.2.1 DNA extraction, library construction, and sequencing

Total genomic DNA was extracted from the 81 samples using the E.Z.N.A. Tissue DNA Kit (Omega Bio-tek, Norcross, GA, USA). The quality and integrity of the extracted DNA were evaluated via 1% agarose gel electrophoresis. Qualified DNA was then digested with restriction enzymes and ligated to P1 adapters containing sample-specific barcodes. The pooled samples were physically sheared to a fragment size range of 300–500 bp, ligated with P2 adapters, and enriched via PCR amplification to construct RAD/ddRAD libraries. Finally, paired-end 150 bp (PE150) sequencing was performed on the Illumina HiSeq platform.

#### 2.2.2 Genomic SNP calling and filtering

Raw sequencing reads in FASTQ format were subjected to rigorous quality control using Trimmomatic 0.36 (parameters: ILLUMINACLIP:adapters.fa:2:30:10 SLIDINGWINDOW:4:15 MINLEN:75) to remove adapter contamination and low-quality bases. Given the absence of a high-quality reference genome, the Stacks software pipeline was employed for *de novo* assembly and SNP calling. Initially, the ustacks program was utilized to align and cluster clean reads into RAD-tags within each sample based on sequence identity, identifying internal heterozygous loci. Subsequently, the populations module was used instead of cstacks to align the RAD-tags across different individuals (allowing a maximum of 2 mismatches) and for specific filtering tasks to identify single-base variations. To minimize artifacts arising from the deep divergence between the two species, strict filtering was applied. The raw SNPs were filtered using VCFtools (maximum missing rate ≤ 0.2 and MAF ≥ 0.05). Furthermore, to meet the assumptions of population structure analyses, Linkage Disequilibrium (LD) pruning was performed using PLINK with the parameters --indep-pairwise 50 10 0.1 (Purcell et al., 2007). Furthermore, to minimize the inclusion of paralogous sequences, which can artificially inflate heterozygosity, an additional filtering step was implemented using a maximum observed heterozygosity threshold of 0.6, yielding high-quality SNP markers.

#### 2.2.3 Data analysis

Based on the highly reliable filtered SNPs, key population genetic parameters, including the inbreeding coefficient (*F*_is_), expected heterozygosity (*H*_e_), observed heterozygosity (*H*_o_), Shannon’s diversity index (*I*), and nucleotide diversity (*π*), were calculated using custom computational scripts. Specifically, to rigorously validate the observed interspecific disparities, the significance of differences in mean genetic diversity indices between *B. tianguii* and *B. andersonii* populations was evaluated using a two-tailed independent samples Wilcoxon rank-sum test in R. Furthermore, pairwise bitwise genetic distances among individuals were calculated using the R package *poppr* to identify distinct multilocus genotypes (MLGs).A phylogenetic tree was constructed based on the neighbor-joining (NJ) method using FastTree 2.1.10. Population genetic structure was inferred using fastSTRUCTURE, and the optimal number of genetic clusters (K) was determined based on the minimum cross-validation (CV) error. Principal Component Analysis (PCA)was conducted using GCTA 1.93.2 to visually assess spatial genetic clustering among individuals. Based on the screened markers, kinship (IBS) analysis was also performed using GCTA software to construct a genetic relationship matrix among pairwise samples, which was visualized as a heatmap. The genetic differentiation coefficients (*F*_ST_) among the 11 populations were calculated utilizing the StAMPP package in R. The resulting *F*_ST_ values were substituted into the standard formula *N*_m_ = (1 - *F*_ST_)/(4*F*_ST_) to estimate the relative proxy intensity of gene flow (*N*_m_). It is important to note that estimating *N*m via Wright’s Island Model assumes an equilibrium between genetic drift and migration. For highly differentiated, fragmented, and declining populations like *B. tianguii*, these equilibrium assumptions are almost certainly violated. Therefore, this formula serves strictly as a relative proxy to compare historical gene flow patterns, rather than providing an absolute measure of contemporary migrants per generation (Whitlock and McCauley, 1999).Finally, a Mantel test was performed using the vegan package in R. Spearman’s correlation coefficients were calculated with permutations for significance testing, generating an Isolation by Distance (IBD) model to evaluate the correlation between genetic and geographic distances. To address the potential bias introduced by uneven sample sizes, we performed a rarefaction analysis. Rarefied Allelic Richness (Ar) was calculated to standardize the sample sizes and ensure robust comparisons of genetic diversity using the R package hierfstat (Goudet, 2005). Differences in Ar between the two species were evaluated using a Kruskal-Wallis rank sum test.

## 3 Results

### 3.1 Sequencing data quality control

GBS was performed on 81 samples using the Illumina HiSeq platform. Following rigorous quality control, the clean reads exhibited Q20 and Q30 scores exceeding 98% and 95%, respectively, indicating excellent sequencing quality. Initial sequence clustering generated 4,787,324 SNPs. After stringent filtering (maximum missing rate ≤ 0.2, minor allele frequency [MAF] ≥ 0.05) to eliminate sequencing errors and repetitive artifacts, a final highly reliable dataset of 23,880 SNPs was retained for subsequent population genetic analyses.

### 3.2 "Genetic diversity analysis of *B. tianguii* and *B. andersonii*

Statistical analysis of the filtered SNP loci was conducted to evaluate the genetic diversity among different populations. The results (**Table 3**) indicate that the average inbreeding coefficient (*F*_is_) values for populations of both species were negative. With the exception of the BA-DT population, which exhibited an *F*_is_ value of 0.166 (*F*_is_ > 0) and an expected heterozygosity (*H*_e_) greater than its observed heterozygosity (*H*_o_), all other populations showed *F*_is_ < 0 and *H*_o_ > *H*_e_. This suggests that outcrossing is the predominant reproductive strategy for both species. Notably, the BT-DT population exhibited an extreme negative *F*_is_ value (-0.954). Subsequent clonal analysis revealed that the pairwise genetic distances among the six BT-DT individuals were exceptionally low (0.18%–0.29%), confirming that they share a single identical multilocus genotype (MLG = 1).

**Table 3:** Genetic diversity of *B. tianguii* and *B. andersonii* populations.

Population	*F*_is_	*H*_o_	*H*_e_	I	π
BT-DT	-0.954	0.118	0.060	0.005	0.066
BT-JG	-0.079	0.103	0.094	0.174	0.102
BT-LHY	-0.636	0.143	0.081	0.045	0.086
BT-LL	-0.212	0.083	0.067	0.106	0.075
BT-NP	-0.220	0.088	0.070	0.124	0.076
BT-TK	-0.290	0.084	0.059	0.072	0.064
BT-WM	-0.271	0.084	0.062	0.092	0.067
**BT-Mean**	-0.380*	0.101**	0.070**	0.088**	0.077**
BA-DT	0.166	0.094	0.111	0.219	0.124
BA-HJD	-0.078	0.109	0.099	0.182	0.107
BA-JX	-0.044	0.105	0.099	0.181	0.110
BA-LHY	-0.101	0.111	0.094	0.150	0.104
**BA-Mean**	-0.014*	0.105**	0.101**	0.183**	0.111**

BT, *B. tianguii*; BA, *B. andersonii*; *indicates a significant difference (*P* < 0.05) and ** indicates an extremely significant difference (*P* < 0.01) in the overall means between BT and BA based on a two-tailed independent samples Wilcoxon rank-sum test.Bold values indicate the overall mean index values for the respective species populations.

**Figure 3:**
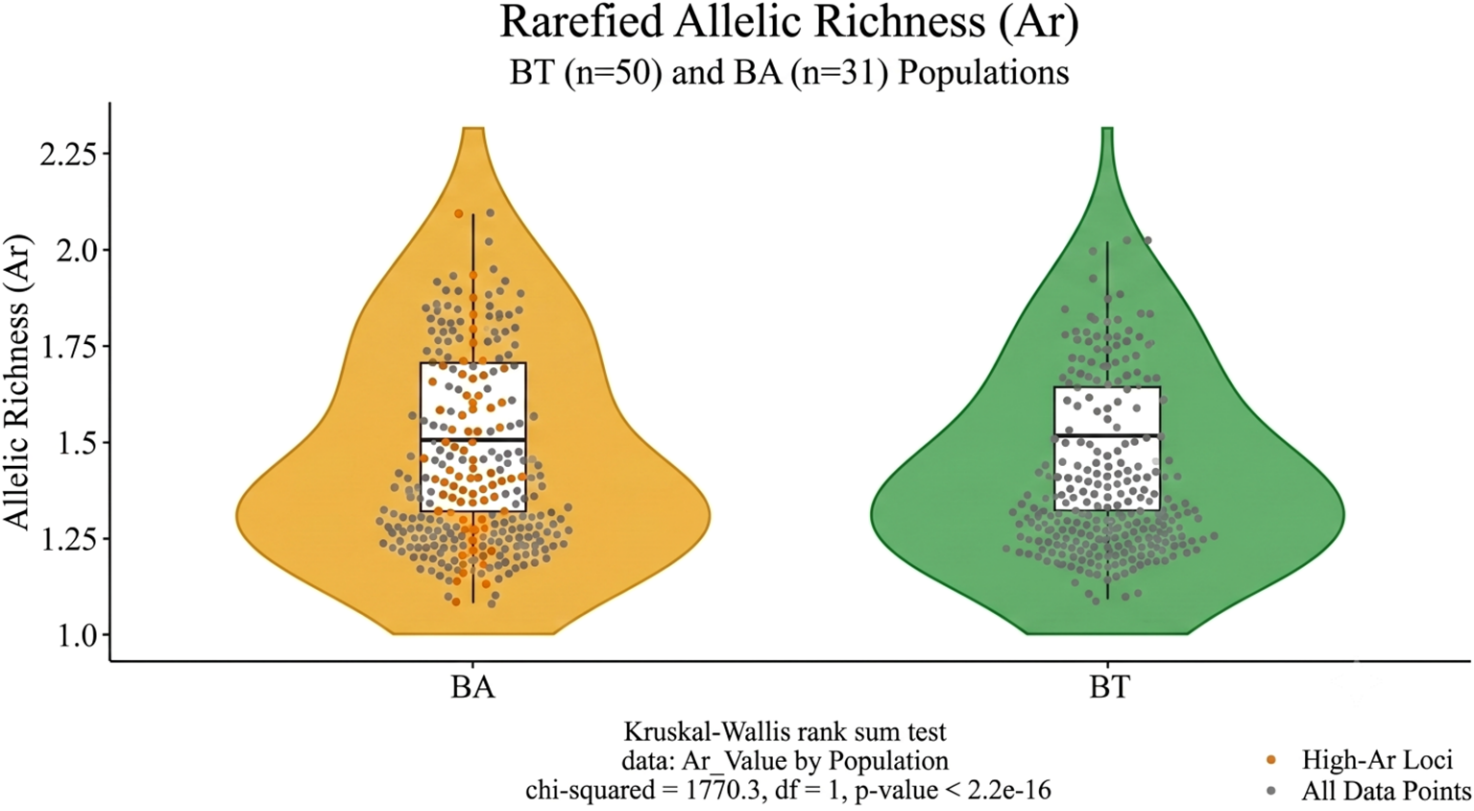
Violin plot of rarefied allelic richness (Ar) between *B. tianguii* (BT) and *B. andersonii* (BA) populations. The Kruskal-Wallis rank sum test indicates a significant difference (chi-squared = 1770.3, df = 1, p-value < 2.2e-16).

For *B. tianguii*, the ranges of *H*_o_, *H*_e_, *I*, and *π* were 0.083–0.143, 0.059–0.094, 0.005–0.174, and 0.064–0.102, respectively, with corresponding mean values of 0.101, 0.070, 0.088, and 0.077. In contrast, the ranges of *H*_o_, *H*_e_, *I*, and *π* for *B. andersonii* were 0.094–0.111, 0.094–0.111, 0.150–0.219, and 0.104–0.124, respectively, with corresponding mean values of 0.105, 0.101, 0.183, and 0.111. Consequently, the mean values of *H*_o_, *H*_e_, *I*, and *π* for the *B. andersonii* populations were 1.04, 1.44, 2.08, and 1.44 times higher than those of the *B. tianguii* populations, respectively. Within *B. tianguii*, the BT-JG population exhibited the highest *H*_e_, *I*, and *π* values, demonstrating distinct genetic diversity differences among populations. Overall, the genetic diversity indices (*H*_o_, *H*_e_, *I*, and *π*) of *B. tianguii* were extremely significantly lower (*P* < 0.01) than those of *B. andersonii* (tested via independent samples Wilcoxon rank-sum test), while its *F*_is_ value was also significantly lower (*P* < 0.05).Furthermore, verification of Rarefied Allelic Richness (Ar) via the Kruskal-Wallis rank sum test revealed a highly significant difference between the two species (chi-squared = 1770.3, df = 1, *P* < 2.2e-16), confirming that the genetic diversity of *B. andersonii* is robustly higher than that of *B. tianguii* even after standardizing sample sizes (**Figure 3**).

### 3.3 Population genetic structure analysis of *B. tianguii* and *B. andersonii*

**Figure 4:**
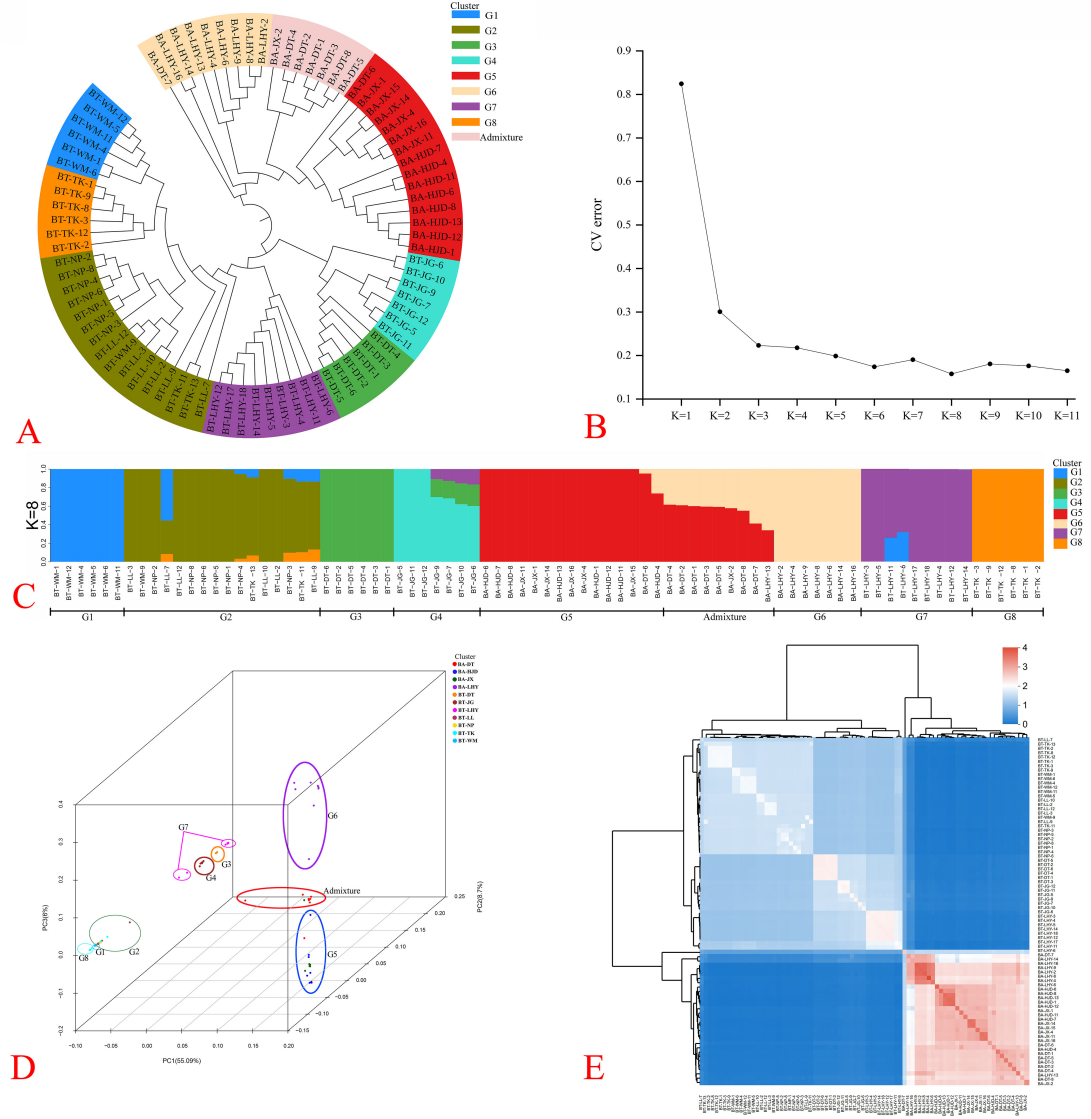
Population genetic structure analysis of *B. tianguii* and *B. andersonii*. **(A)** Phylogenetic tree; **(B)** Cross-validation error curve for K values; **(C)** STRUCTURE bar plot; **(D)** Principal component analysis (PCA)plot; **(E)** Kinship heatmap.

The fastStructure analysis indicated that based on the minimum cross-validation (CV) error, the optimal K value was 8 (**Figure 4B**), suggesting that the 81 samples were best divided into eight genetic clusters (**Figure 4C**). Based on a probability threshold of Q ≥ 0.70 (i.e., an accession with a membership score > 0.70 is reliably assigned to a specific population, while those with scores between 0.50 and 0.70 are considered to have partial admixture), the 81 samples were classified into eight subpopulations (G1–G8) and one admixed group (**Figure 4C**). Notably, the BT (*B. tianguii*) and BA (*B. andersonii*) populations were completely separated, indicating an absence of natural hybridization between the two species. The BT population was divided into six subpopulations (G1–G4, G7, and G8). Specifically, G1, G3, G4, G7, and G8 comprised partial or all individuals from the BT-WM, BT-DT, BT-JG, BT-LHY, and BT-TK populations, respectively. However, the G2 subpopulation contained partial samples from four populations (BT-LL, BT-WM, BT-NP, and BT-TK), implying a certain degree of gene flow among them. Conversely, the BA population was divided into G5 (containing all BA-HJD and partial BA-JX samples), G6 (containing all BA-LHY samples), and an admixed group (containing all BA-DT and partial BA-JX samples). This suggests distinct gene flow between the BA-JX population and both the BA-DT and BA-HJD populations.

Phylogenetic analysis revealed that the 81 samples were divided into two major clades corresponding to *B. tianguii* (BT) and *B. andersonii* (BA), which were further subdivided into nine groups (G1–G8 and one Admixture group) (**Figure 4A**). The BT clade contained six subpopulations, while the BA clade consisted of two subpopulations and one admixed group, which is highly consistent with the STRUCTURE analysis results. Principal component analysis (PCA) showed that the first three principal components (PC1, PC2, and PC3) accounted for 55.09%, 8.70%, and 6.00% of the total variance, respectively. These three principal components similarly separated the BT and BA populations with high clarity. The BT population was partitioned into six subpopulations (G1–G4, G7, and G8), and the BA population into three groups (G5, G6, and an Admixture group) (**Figure 4D**). The close clustering of G1, G2, and G8 indicated a close genetic relationship among the BT-LL, BT-WM, BT-NP, and BT-TK populations. Similarly, the tight clustering of G3, G4, and G7 suggested close affinities among the BT-DT, BT-JG, and BT-LHY populations. Meanwhile, the three BA populations exhibited relatively strong clustering, reflecting robust inter-population gene flow. Kinship analysis evaluated the genetic relatedness among populations based on heatmap color intensity. Redder hues indicate a closer genetic relationship among samples; a predominantly red heatmap among multiple samples within the same population suggests they may constitute a closely related family group. Conversely, bluer hues indicate greater genetic distance. In this study, both inter- and intra-population kinships in *B. tianguii* were weaker than those in *B. andersonii* (**Figure 4E**).

### 3.4 Population genetic differentiation and gene flow analysis of *B. tianguii* and *B. andersonii*

**Figure 5:**
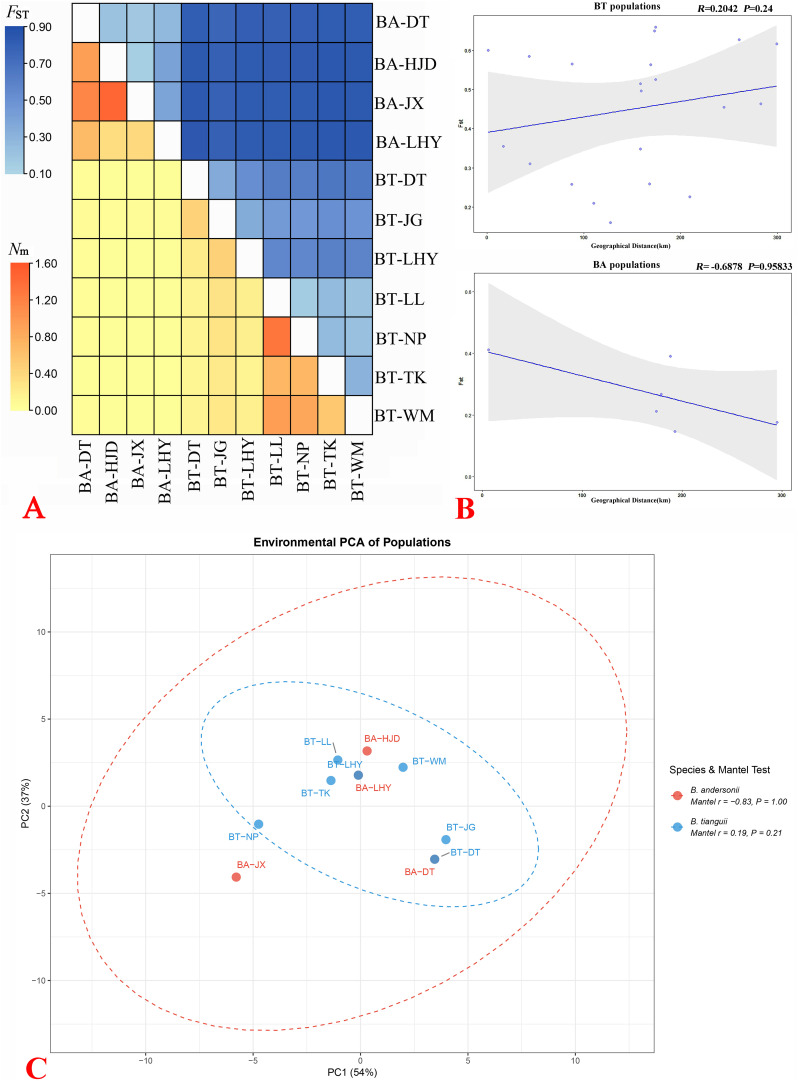
Genetic differentiation, gene flow, and isolation effects in *Bulbophyllum andersonii* and *B. tianguii* populations. **(A)** Heatmap of pairwise genetic differentiation (*F*_ST_, upper right, blue) and gene flow (*N*_m_, lower left, orange). **(B)** Isolation by distance (IBD)analysis. Scatter plots show Mantel test results for correlation between genetic and geographical distances (grey areas represent 95% confidence intervals). **(C)** Environmental Principal Component Analysis (PCA) of populations. The legend shows Mantel test results for isolation by environment (IBE). BA, *B. andersonii*; BT, *B. tianguii*; *F*_ST_, genetic differentiation index; *N*_m_, gene flow.

Analysis of genetic differentiation and gene flow (**Table 4**; **Figure 5A**) revealed that the genetic differentiation index (*F*_ST_) among *B. tianguii* populations ranged from 0.160 to 0.659, with a mean of 0.450. The majority (85.7%) of pairwise population comparisons exhibited *F*_ST_ > 0.25, indicating high genetic differentiation among most populations. Although gene flow (*N*_m_) values were mathematically derived from *F*_ST_ and serve strictly as a relative proxy under island model assumptions rather than absolute migrant counts, to provide a relative reference, the estimated *N*_m_ among *B. tianguii* populations averaged 0.411, consistent with the observed high differentiation pattern. For *B. andersonii*, the *F*_ST_ values among populations ranged from 0.147 to 0.411, with an average of 0.268. The calculated *N*_m_ values for *B. andersonii* (mean 0.830) were generally higher than those of *B. tianguii*, reflecting more frequent historical genetic exchange. Between the two species, the interspecific *N*_m_ values ranged from 0.045 to 0.076, with an average of 0.054, indicating an almost complete absence of historical gene flow between the populations of the two species.

**Table 4:** Gene flow (*N*_m_) and genetic differentiation coefficient (*F*_ST_) among populations of *B. tianguii* and *B. andersonii*.

*N*_m_/*F*_ST_	BA-DT	BA-HJD	BA-JX	BA-LHY	BT-DT	BT-JG	BT-LHY	BT-LL	BT-NP	BT-TK	BT-WM
BA-DT	——	0.212	0.177	0.268	0.815	0.766	0.806	0.798	0.807	0.822	0.817
BA-HJD	0.930	——	0.147	0.411	0.838	0.797	0.829	0.825	0.831	0.844	0.839
BA-JX	1.163	1.452	——	0.391	0.838	0.792	0.826	0.823	0.829	0.843	0.838
BA-LHY	0.683	0.359	0.390	——	0.842	0.794	0.829	0.829	0.833	0.847	0.843
BT-DT	0.057	0.048	0.049	0.047	——	0.355	0.525	0.627	0.617	0.659	0.649
BT-JG	0.076	0.063	0.066	0.065	0.454	——	0.348	0.455	0.463	0.515	0.496
BT-LHY	0.060	0.052	0.053	0.052	0.226	0.469	——	0.565	0.563	0.600	0.585
BT-LL	0.063	0.053	0.054	0.052	0.149	0.300	0.193	——	0.160	0.258	0.209
BT-NP	0.060	0.051	0.052	0.050	0.155	0.290	0.194	1.309	——	0.259	0.226
BT-TK	0.054	0.046	0.047	0.045	0.130	0.235	0.167	0.719	0.716	——	0.310
BT-WM	0.056	0.048	0.048	0.047	0.135	0.254	0.177	0.948	0.858	0.557	——

The values in the lower triangle represent the gene flow intensities (*N*_m_) among populations; the values in the upper triangle represent the genetic differentiation coefficients (*F*_ST_) among populations.

The Mantel test results (**Figure 5B**) demonstrated no significant correlation between genetic differentiation and geographic distance for either *B. tianguii* (R = 0.204, *P* = 0.240) or *B. andersonii* (R = -0.688, *P* = 0.958). This indicates that geographic distance is not the primary driver of the genetic differentiation observed within the populations of these two orchid species. Furthermore, to explicitly assess the impact of macroclimatic factors on genetic divergence, we conducted an Environmental Principal Component Analysis (PCA) and Isolation by Environment (IBE) Mantel tests based on 19 standard bioclimatic variables. The Environmental PCA (**Figure 5C**) revealed that *B. andersonii* occupies an exceptionally broad macroclimatic niche (indicated by the large dispersed ellipse), reflecting its robust environmental adaptability. In striking contrast, the macroclimatic niche of *B. tianguii* is remarkably narrow and is completely nested within that of *B. andersonii*. This nesting phenomenon perfectly explains their sympatric distribution in certain locations, as *B. tianguii* does not occupy a unique macroclimatic refuge unavailable to *B. andersonii*. Moreover, the IBE Mantel tests demonstrated that there is no significant correlation between genetic differentiation and macroclimatic environmental distance for either *B. tianguii* (r = 0.19, *P* = 0.21) or *B. andersonii* (r = -0.83, *P* = 1.00).

## 4 Discussion

### 4.1 Genetic diversity

The level of genetic diversity has a significant impact on the long-term survival of endangered plants (Markert et al., 2010). Generally, higher genetic diversity correlates with a stronger adaptive capacity of a species to habitat changes, whereas lower diversity indicates weaker adaptability. Genetic parameters based on SNP loci, such as *π*, *H*_o_, and *H*_e_, are critical indicators for measuring the level of population genetic diversity (Jakobsson et al., 2007). Among these, *π* serves as a comprehensive indicator, with a higher *π* value reflecting greater genetic diversity (Catchen et al., 2013**;** Tichkule et al., 2021). This study revealed that the genetic diversity of *B. tianguii* populations (*π* = 0.077, *H*_o_ = 0.101, *H*_e_ = 0.070) was lower than the average for terrestrial orchids (*H*_e_ = 0.119) (Case, 2002). It was also significantly lower than that of other endangered species within the genus *Bulbophyllum* and the family Orchidaceae, such as *Bulbophyllum exaltatum* (*H*_o_ = 0.0832, *H*_e_ = 0.266) and *Bulbophyllum involutum* (*H*_o_ = 0.0871, *H*_e_ = 0.267, assessed by Isozyme analysis) (Ribeiro et al., 2008), as well as *Cymbidium tortisepalum* (*H*_o_ = 0.619, *H*_e_ = 0.653, assessed by nuclear Simple Sequence Repeats, nSSR) (Zhao et al., 2017), *Geodorum eulophioides* (*H*_o_ = 0.1822, *H*_e_ = 0.1553, assessed by GBS) (Zhu et al., 2023), and *Dendrobium loddigesii* (*H*_o_ = 0.3446, *H*_e_ = 0.3588, assessed by SSR)(Ding et al., 2012). Although cross-study numerical comparisons must be interpreted with caution due to the different mutational rates and the biallelic nature of most SNPs. Since SNPs are predominantly biallelic, their maximum theoretical heterozygosity is capped at 0.5. In contrast, multi-allelic markers like SSRs can yield heterozygosity values approaching 1.0 due to their hypermutability. Consequently, direct absolute numerical comparisons across different marker types can exaggerate the biological reality. Nevertheless, when compared within the context of similar marker systems or generalized trends, the relative trend unmistakably highlights the genuinely low genetic diversity of *B. tianguii*, implying relatively poor adaptability to its habitat and underscoring the need for further comparative research on its genetic diversity.

Relevant studies have demonstrated that endangered, narrowly distributed species typically exhibit lower genetic diversity than their widely distributed congeners. For instance, the narrowly distributed *Eriogonum soredium* (*H*_e_ = 0.28) exhibits lower genetic diversity than the widely distributed *Eriogonum shockleyi* (*H*_e_ = 0.32) (Lemon and Wolf, 2018); the genetic diversity of *Carpinus putoensis* Cheng is lower than that of *Carpinus laxiflora* (Siebold et Zucc.)Blume (Zhang et al., 2011**;** Ahn et al., 2019); and the genetic diversity of *Ardisia violacea* is lower than that of its widely distributed congener *Ardisia crenata* Sims (Zhou et al., 2023**;** Luo et al., 2021). In this study, the overall genetic diversity of the seven *B. tianguii* populations (*π* = 0.077) was significantly lower (*P* < 0.01) than that of its widely distributed congener, *B. andersonii* (*π* = 0.111). It is generally believed that the lower genetic diversity in rare or endangered species is primarily caused by their narrow distribution range, limited population size, pronounced population isolation, and the need to adapt to a specific and uniform habitat (Frankham et al., 2012). This conclusion was corroborated during the field surveys of this study: the individual population area (1–56 m²) and population size (25–615 individuals) of *B. tianguii* were substantially smaller than those of *B. andersonii* (population area of 50–120 m² and 850–1500 individuals). Notably, the BT-DT population (*π* = 0.066) currently survives in an area of only about 1 m², consisting of a mere 25 plants. Extremely small populations further accelerate the rate of genetic drift, generally leading to the fixation or loss of certain alleles after one or a few generations, thereby exacerbating inter-population genetic variation and reducing overall genetic diversity (Freeland, 2005). This is likely a crucial reason why the genetic diversity of *B. tianguii* is significantly lower than that of *B. andersonii*. It is worth noting that smaller sample sizes tend to underestimate rare alleles and bias diversity estimates downward. In our study, *B. andersonii* had a smaller sample size (n=31) compared to *B. tianguii* (n=50), yet it still exhibited significantly higher genetic diversity. This implies that the true genetic diversity of *B. andersonii* might be even higher. Our supplementary rarefaction analysis, which standardized sample sizes, further verified this robust disparity in Rarefied Allelic Richness (Ar).

Plant genetic diversity is influenced by multiple factors. In addition to geographical distribution range (Zhang et al., 2015)and population size, it is intricately linked to various life-history traits, including life form, breeding system, growth habits, and evolutionary history (Gitzendanner and Soltis, 2000**;** Nybom and Bartish, 2000). In particular, the breeding system is a critical determinant of species genetic diversity (Ge et al., 1997). Jiang et al. (2020) investigated the pollination biology of *B. tianguii* in the Laowuji sinkhole of the Yachang Nature Reserve and confirmed that its fruiting rate (2.5%)is far below the average level for orchids (20.7%) (Hu et al., 2018). Furthermore, Jiang et al. (2025) discovered that the sole pollinator of *B. tianguii* is the flesh fly (*Sarcophaga carnaria*), which exhibits an extremely low visitation frequency (0.005 visits/flower/h). They also noted that the breeding system of *B. tianguii* demonstrates both self- and cross-compatibility. While self-pollinating orchids typically display lower genetic diversity within populations, the *B. tianguii* populations in this study exhibited an outcrossing tendency (*F*_is_ < 0). Notably, some populations exhibited extreme negative *F*_is_ values (e.g., BT-DT = -0.954). This phenomenon of abnormally high heterozygosity can be primarily attributed to the synergistic effects of bioinformatics artifacts and the species’ compensatory reproductive strategies. In severe karst habitats, constrained by extremely low pollination frequencies and fruiting rates, *B. tianguii* might rely heavily on vegetative propagation (e.g., rhizome division) as a survival mechanism. If a small 1m² area is dominated by only one or a few clonal lineages, extensive clonal reproduction can lead to fixed heterozygosity, strongly shifting *F*_is_ values toward extreme negatives. To definitively distinguish this biological reality from potential assembly artifacts (e.g., artificial merging of paralogous loci), we calculated pairwise bitwise genetic distances among the six BT-DT individuals using the R package poppr. The extremely low genetic distances (0.18%–0.29%) confirm that they represent a single multilocus genotype (MLG), proving that extensive vegetative propagation is the true biological driver of the extreme negative *F*_is_ value. The hypothesis of extensive clonal reproduction in the BT-DT population is further corroborated by our kinship analysis (**Figure 4E**), which revealed localized, extremely close genetic relatedness among individuals within its restricted 1 m² area. Consequently, it can be further inferred that severe pollination limitation and the resulting extremely low fruiting rate not only force the species to over-rely on clonal growth but also represent the primary root cause of its reproductive barriers and propagation difficulties. In contrast, although the sole pollinator of *B. andersonii* is *Cerodontha curvicornis*, its visitation frequency is remarkably high (20 visits/flower/h), and its natural fruiting rate can reach 26.06% (He et al., 2024), substantially outperforming *B. tianguii*. Both efficient pollination and a high natural fruiting rate actively facilitate the maintenance of higher genetic diversity in *B. andersonii*. In conclusion, a constrained reproductive rate is another key factor contributing to the markedly lower genetic diversity of *B. tianguii* compared to *B. andersonii*.

### 4.2 Genetic differentiation and genetic structure

Genetic differentiation is typically influenced by biological characteristics such as habitat features and adaptability, pollination biology, seed dispersal, and gene flow, it is a crucial indicator for measuring the extent of genetic differentiation among populations (Liu et al., 2022). Generally, a higher *F*_ST_ value indicates a more distant genetic relationship. According to Wright (1965) classification criteria for *F*_ST_, the vast majority of *B. tianguii* populations in this study exhibited *F*_ST_ values exceeding 0.25, indicating a state of extremely high genetic differentiation. Based on our findings, The degree of genetic differentiation among *B. tianguii* populations was substantially higher than the average for terrestrial orchids (*F*_ST_ = 0.161) (Forrest et al., 2004). It was also much higher than that of other endangered species within the genus *Bulbophyllum*, such as *Bulbophyllum exaltatum* (*F*_ST_ = 0.230, *N*_m_ = 0.755)and *B. involutum* (*F*_ST_ = 0.232, *N*_m_ = 0.608) (Ribeiro et al., 2008), as well as the genus *Cypripedium* in the Orchidaceae family (*F*_ST_ = 0.150) (Chung et al., 2009). Hamrick, (1987) noted that a gene flow level of *N*_m_ > 1 can partially counteract the exacerbating effect of genetic drift on differentiation; thus, an *N*_m_ > 1 indicates strong gene exchange among populations. Results demonstrate that the genetic differentiation among *B. tianguii* populations is significantly higher than that of *B. andersonii*.

This disparity may be attributed to the habitat specificity of *B. tianguii*, compared to the stronger habitat adaptability and broader distribution range of *B. andersonii*. It is well known that orchids rely on symbiotic fungi to facilitate seed germination. However, the fungal communities in the roots of *B. tianguii* vary significantly across different habitats; the number of operational taxonomic units (OTUs) in humus and rocky habitats is significantly higher than in epiphytic (tree-trunk) habitats, with humus exhibiting the highest mycorrhizal fungal diversity (Liang et al., 2022). This explains why *B. tianguii* predominantly prefers to grow epiphytically on the cliffs of karst gorges, understory rocks, mountaintop rocks, or humus-rich tree stems (Lang and Luo, 2007). Its profound habitat specificity (Chai et al., 2022)restricts gene exchange and dispersal reproduction among populations, potentially leading to further population declines. Conversely, *B. andersonii* achieves high seed germination rates in various specialized epiphytic habitats, such as rock walls, crevices, tree trunks, and humus layers, demonstrating robust adaptability to diverse environments (Wu et al., 2024**;** Hietz et al., 2022). This adaptability creates favorable conditions for population expansion and gene exchange. During field surveys, we observed that *B. tianguii* and *B. andersonii* are sympatrically distributed in two locations: the Orchid Garden of the Yachang Nature Reserve and the Dongtian site at the Xiazhai Station of the Mulun Nature Reserve in Huanjiang County, Guangxi. The habitats in both locations are either rocky or epiphytic on trees. However, the population size of *B. andersonii* (Orchid Garden: 850 plants across 50 m²; Dongtian: 1050 plants across 75 m²) far exceeds that of *B. tianguii* (Orchid Garden: 125 plants across 12 m²; Dongtian: 25 plants across 1 m²). This clearly indicates that under sympatric conditions, the reproductive capacity of *B. tianguii* is significantly weaker than that of *B. andersonii*, a finding closely linked to the species’ habitat adaptability. Generally, the more pronounced a species’ habitat specificity, the weaker its adaptability, which subsequently leads to lower gene flow and higher genetic differentiation among populations (Zhu et al., 2023). This aligns with our finding that the genetic differentiation of *B. tianguii* is much higher than that of *B. andersonii*. Furthermore, most extant *B. tianguii* populations are currently distributed outside nature reserves. Driven by economic interests, human over-exploitation and habitat destruction have reduced the effective population size and compromised connectivity among populations. This intensifies genetic drift and inbreeding, decreases species genetic diversity, and, under restricted gene flow, further elevates genetic differentiation among populations (Cai et al., 2021).

Genetic structure not only reflects the evolutionary history of a population but also indicates its future evolutionary potential to some extent (McDonald and Linde, 2002). Key factors influencing population genetic structure include gene flow, genetic mutation, genetic drift, habitat fragmentation, and anthropogenic disturbances (Su et al., 2017). Our results demonstrated that geographically proximate populations (e.g., BT-LHY and BT-TK) could exhibit weak gene exchange, whereas geographically distant populations (e.g., BT-NP and BT-LL) maintained relatively strong connections. This aligns with the Mantel test, confirming no significant correlation between genetic differentiation and geographic distance among *B. tianguii* populations. STRUCTURE analysis also indicated a closer genetic relationship and relatively stronger gene exchange among four specific populations: BT-LL, BT-WM, BT-NP, and BT-TK. Meanwhile, the BT-DT, BT-JG, and BT-LHY populations were more closely related to each other. This pattern may emerge because the habitats of the BT-DT, BT-JG, and BT-LHY populations are more similar—mostly located on rocks or tree trunks in the understory of the mid-to-upper parts of karst mountains at lower altitudes. Conversely, the BT-LL, BT-WM, BT-NP, and BT-TK populations are predominantly situated on sinkhole cliffs at higher altitudes. Similar habitats are more conducive to population expansion and reproduction, facilitating closer genetic relationships. Additionally, the presence of karst mountain barriers between the BT-LHY and BT-TK populations may form a natural obstacle to the dispersal of *B. tianguii* pollen and seeds, thereby impeding gene exchange and resulting in high genetic differentiation (Hermansen et al., 2017).

A similar pattern was observed in *B. andersonii*, where the Mantel test confirmed the lack of isolation by distance, with distant populations sometimes maintaining stronger gene exchange than adjacent ones. However, the overall gene flow among *B. andersonii* populations was stronger than that of *B. tianguii*. Furthermore, the phylogenetic relationships indicated that both inter- and intra-population relationships were weaker in *B. tianguii* compared to *B. andersonii*. This is linked to the limited dispersal capacity of *B. tianguii* and ongoing habitat fragmentation, both of which restrict inter-population gene flow. Our dual Mantel test results present a compelling “double negative”: neither geographic distance (IBD) nor macroclimatic environmental distance (IBE) significantly correlates with the high genetic differentiation observed in *B. tianguii*. Furthermore, the Environmental PCA visually confirms that the macroclimatic niche of *B. tianguii* is completely nested within the broader niche of *B. andersonii*. Since regional-scale macroclimatic variables (such as temperature and precipitation) fail to explain the restricted gene flow, what is the true driver of this extreme genetic divergence? We hypothesize that microhabitat specificity acts as a primary driving factor. Unlike *B. andersonii*, which exhibits robust adaptability to various substrates, *B. tianguii* is characterized by extreme habitat specificity, strictly requiring specific calcareous substrates, particular humus layers, and highly specialized symbiotic mycorrhizal fungal communities for successful seed germination (Liang et al., 2022). These profound microhabitat specificities, which are entirely invisible to broad-scale macroclimatic grid data, may act as formidable ecological barriers that severely restrict inter-population seed and pollen dispersal. However, this inferential chain requires direct experimental validation. Furthermore, it is highly probable that other factors, such as complex demographic histories (e.g., historical genetic bottlenecks and subsequent genetic drift fixing alleles in small isolated populations) and restricted pollinator activity (e.g., specific fly pollinators with limited flight distances), have collaboratively shaped the observed high-differentiation patterns. Coupled with the accelerated genetic drift inherent in its extremely small and fragmented populations, these combined pressures have ultimately sculpted the “high differentiation, low gene flow” genetic landscape of *B. tianguii*.

### 4.3 Endangered mechanisms and conservation strategies for *B. tianguii*

It is generally believed that the causes of species endangerment mainly encompass two aspects: internal biological characteristics (such as extremely low genetic diversity) and external ecological stresses (such as habitat destruction and environmental changes) (Falk and Holsinger, 1991**;** Schemske et al., 1994). These two dimensions must be fully integrated when formulating comprehensive species conservation plans. Given the extremely low genetic diversity of *B. tianguii*, its inherent adaptive capacity to environmental fluctuations is exceptionally poor. Consequently, any alteration in its microhabitat can easily lead to a further loss of genetic diversity, potentially pushing the species to the brink of extinction. This extremely low diversity may not only be the result of recent anthropogenic habitat destruction but also reflects its evolutionary history, suggesting that it may have experienced a severe historical genetic bottleneck or a strong founder effect during speciation. Therefore, conservation efforts should adopt a holistic strategy: prioritizing *in situ* conservation, supplemented by artificial propagation and targeted wild reintroduction. Specifically, the *in situ* conservation of original habitats remains the primary task for preserving its extant genetic diversity. For populations distributed outside established nature reserves (i.e., the BT-LL, BT-NP, and BT-WM populations), there is an urgent need to establish conservation micro-reserves to protect the surviving plants and their local habitats, thereby maintaining the species’ genetic viability. Among the seven populations studied, the BT-JG population exhibited the highest level of genetic diversity; therefore, it should be prioritized as a core target for conservation efforts. Furthermore, measures for the artificial propagation and population reintroduction of *B. tianguii* must be strengthened. When implementing *ex situ* conservation and artificial reintroduction, it is insufficient to merely select populations; we must explicitly identify core seed-source individuals within genetically diverse populations (such as BT-JG and BT-LHY) to capture the maximum available standing genetic variation. Moreover, establishing a self-sustaining reintroduced population requires a rigorous evaluation of the minimum viable population (MVP) size to effectively buffer against future genetic drift and inbreeding depression. The determination of reintroduction locations is equally critical. These sites must not only be selected based on strict microhabitat matching—ensuring the availability of necessary calcareous substrates and highly specific symbiotic mycorrhizal fungi (McCormick and Jacquemyn, 2014)—but should also be strategically placed at appropriate geographical distances from native ranges. This spatial planning ensures the new sites fall within the species’ historical distribution while preventing the potential spread of localized pathogens. However, when mixing seeds or cross-pollinating these source individuals, conservationists must carefully evaluate the genetic compatibility between source and recipient lineages to prevent potential outbreeding depression and the disruption of local adaptations (Frankham et al., 2011). Ultimately, scientifically expanding the spatial distribution and restoring the sizes of existing populations will help mitigate the trend of population decline, protect the species’ invaluable gene pool, and prevent the irreversible loss of genetic resources. In addition, local public awareness and environmental education campaigns should be concurrently enhanced to minimize anthropogenic disturbances, thereby preventing the human-induced depletion of wild populations and the degradation of their fragile natural habitats.

### 4.4 Limitations of the study

This study has several limitations that warrant consideration. First, the uneven sample sizes between the two species (50 individuals across seven populations for *B. tianguii* versus 31 individuals across four populations for *B. andersonii*) may introduce statistical biases, potentially overestimating genetic differentiation (*F*_ST_) and destabilizing genetic diversity estimates. However, our rarefaction analyses (Rarefied Allelic Richness) effectively mitigated this concern, confirming the robustness of the observed comparative diversity patterns. Second, performing a *de novo* assembly that merges two distinct species without a reference genome runs the risk of increasing the proportion of erroneous SNPs, even with stringent filtering parameters in place. Finally, we retained the six clonal individuals (MLG = 1) from the critically small BT-DT population in our structural analyses (PCA and STRUCTURE). While this approach may subtly skew local allele frequencies, it serves to accurately capture the true biological condition of this population—specifically, a profound reliance on vegetative propagation driven by severe pollination limitations, which has culminated in an inherently depressed genetic state.

## 5 Conclusions

In conclusion, this study provides the first genome-wide assessment using GBS technology to uncover the critically low genetic diversity and high population differentiation of the endangered *B. tianguii* compared to its widespread congener *B. andersonii*. While limited by uneven sampling sizes and the absence of a reference genome, our findings highlight that small population size, specialized habitat, and restricted gene flow are key drivers of its endangerment. To safeguard its remaining gene pool, immediate *in situ* conservation is imperative, especially for the relatively diverse BT-JG population. Furthermore, future *ex situ* conservation and reintroduction efforts should critically consider microhabitat matching—particularly integrating joint analyses with root-associated mycorrhizal fungal communities—while monitoring for potential outbreeding depression.

## Data Availability

The original contributions presented in the study are publicly available. This data can be found here: NCBI, PRJNA1483386.
